# Long-term dopaminergic therapy improves spoken language in de-novo Parkinson’s disease

**DOI:** 10.1007/s00415-025-13070-8

**Published:** 2025-04-17

**Authors:** Martin Subert, Tereza Tykalova, Michal Novotny, Ondrej Bezdicek, Petr Dusek, Jan Rusz

**Affiliations:** 1 Department of Circuit Theory, Faculty of Electrical Engineering, Czech Technical University in Prague, Technická 2, Praha 6, 160 00 Prague, Czech Republic; 2 Department of Neurology and Centre of Clinical Neuroscience, First Faculty of Medicine, Charles University and General University Hospital, Prague, Czech Republic; 3 ARTORG Center for Biomedical Engineering Research, University of Bern, Bern, Switzerland

**Keywords:** Linguistic analysis, Levodopa, Natural language processing, Speech, Discourse

## Abstract

**Background and objectives:**

The impact of dopaminergic medication on language in Parkinson’s disease (PD) remains poorly understood. This observational, naturalistic study aimed to investigate the effects of long-term dopaminergic therapy on language performance in patients with de-novo PD based on a high-level linguistic analysis of natural spontaneous discourse.

**Methods:**

A fairy-tale narration was recorded at baseline and a 12-month follow-up. The speech samples were automatically analyzed using six representative lexical and syntactic features based on automatic speech recognition and natural language processing.

**Results:**

We enrolled 109 de-novo PD patients compared to 68 healthy controls. All subjects completed the 12-month follow-up; 92 PD patients were on stable dopaminergic medication (PD-treated), while 17 PD patients remained without medication (PD-untreated). At baseline, the PD-treated group exhibited abnormalities in syntactic domains, particularly in sentence length (*p* = 0.018) and sentence development (*p* = 0.042) compared to healthy controls. After 12 months of dopaminergic therapy, PD-treated showed improvements in the syntactic domain, including sentence length (*p* = 0.012) and sentence development *(p* = 0.030). Of all PD-treated patients, 37 were on monotherapy with dopamine agonists and manifested improvement in sentence length (*p* = 0.048), while 32 were on monotherapy with levodopa and had no language amelioration. No changes in language parameters over time were seen in both the PD-untreated group and healthy controls.

**Discussion:**

Initiation of dopaminergic therapy improved high-language syntactic deficits in de-novo PD, confirming the role of dopamine in cognitive-linguistic processing. Automated linguistic analysis of spontaneous speech via natural language processing can assist in improving the prediction and management of language deficits in PD.

## Introduction

Parkinson’s disease (PD) is a progressive neurodegenerative disorder predominantly characterized by motor impairments [1]. In addition to motor deficits, PD patients commonly manifest with cognitive decline, with up to 40% of de-novo PD presenting with mild cognitive impairment (MCI) [2] and up to 80% of PD developing dementia throughout the disease [3]. Language is a crucial component of cognitive functions and a significant predictor of potential cognitive decline in various neurodegenerative disorders [4]. Indeed, multiple language deficiencies have been reported in PD patients, including decreased informational content, syntax complexity, and grammatical performance [5–7].

Dopaminergic therapy is widely used in PD to compensate for dopamine deficiency, which causes dysfunction of basal ganglia-cortical circuits. While this therapy is primarily given to improve motor symptoms, dopaminergic transmission plays a crucial role not only in motor control but also in cognitive and language functions [8]. However, the impact of levodopa on language performance remains poorly understood [9–13]. While the available evidence tends to support the positive effect of dopaminergic therapy on language, previous studies are mostly limited due to small sample sizes, heterogeneity of the studied population, and analysis of only the short-term medication effects [9–11]. In addition, most of the studies relied on the evaluation of simple verbal fluency tests [10–13], which are insufficient to capture the complexities of language changes in real-life functioning.

Therefore, this study investigated the long-term effect of dopaminergic therapy introduction on language functions in de-novo, untreated PD using a complex analysis of lexical and syntactic structures based on natural language processing. We hypothesized that long-term dopaminergic therapy would improve language abilities in PD.

## Subjects and methods

### Standard protocol approvals, registrations, and patient consents

The study was approved by the Ethics Committee of the General University Hospital in Prague, Czech Republic, and has therefore been performed in accordance with the ethical standards established in the 1964 Declaration of Helsinki. All participants provided written, informed consent to the neurological examination and recording procedure.

### Study design and participants

Between 2015 and 2023, we prospectively enrolled patients with de-novo, drug-I PD at Charles University and General University Hospital in Prague, Czechia. The recruitment was part of a longitudinal project, “biomarkers in PD (BIO-PD)”; the detailed protocol of this project has been described previously [14]. The diagnosis for all patients was established based on the Movement Disorder Society’s clinical diagnostic criteria for PD [15]. The patients were excluded if they had previously received antiparkinsonian treatment, had significant neurological or communication disorders not related to PD, and were non-native Czech speakers. All participants underwent follow-up assessment after a 12-month period. Those on stable dopaminergic therapy consisting mainly of levodopa and/or dopamine agonists were retested in a practically defined “off”-medication state in the morning after a 12-h withdrawal from levodopa or a 24-h withdrawal from dopamine agonists, ensuring the exclusion of acute medication effects. Dopaminergic doses were recalculated to levodopa dose equivalents [16]. In addition, a control group of age-, sex-, and education-matched Czech-native healthy individuals without MCI, neurological disorders, or communication disorders was recruited and retested after 12 months.

### Clinical examination

At baseline, each participant underwent a comprehensive clinical evaluation, which included personal and medical history, history of drug, substance intake, and current drug use. The motor symptoms were quantitatively assessed using the Movement Disorder Society–Unified Parkinson Disease Rating Scale (MDS-UPDRS) part III [17]. From the MDS-UPDRS part III, composite scores including bradykinesia (sum of items 3.4–3.8 and 3.14), rigidity (sum of items 3.3), tremor (sum of items 3.15–3.18), and postural instability/gait difficulty (PIGD; sum of items 3.9–3.13) were also calculated. Cognitive function was assessed using the Montreal Cognitive Assessment (MoCA) [18]. From MoCA, a composite Language score (Repeat and Fluency) was computed. As a part of the MoCA Language score, the phonemic score fluency (as many words starting with “K” in one minute) was used separately in further analyses. The presence of MCI was determined as a MoCA score lower than 1.5 standard deviation below the age-related normative mean [18]. Neuropsychiatric assessment included depressive symptoms using Beck Depression Inventory-II [19, 20], and State-Trait Anxiety Inventory X1/X2 (STAI-X1, STAI-X2) [21]. The disease duration was estimated from the self-reported occurrence of the first motor symptoms. Motor, cognitive, and neuropsychiatric assessments were repeated at the 12-month follow-up.

### Speech assessment

The participants underwent a speech examination guided by a professional. Each participant was instructed to narrate a fairy tale for approximately 90 s. The narration task was recorded in a single session in a quiet room, using a head-mounted condenser microphone (Beyerdynamic Opus 55, Heilbronn, Germany) positioned approximately 5 cm from the subject’s mouth. The recordings utilized 48 kHz sampling and 16-bit resolution. The average duration of the narration task was 106 s (standard deviation [SD] 22) and contained an average of 168 words (SD 40). The narration task was preferred due to its better sensitivity in reflecting cognitive language abnormalities in PD compared to monologue on a freely chosen topic [22] as well as its suitability in predicting the development of cognitive impairment in prodromal synucleinopathy [23].

### Speech transcription and annotation

Each recording was automatically transcribed using the automatic speech recognition system, Whisper [24], with the latest multilingual model large-v3 [25], which reports a word error rate of 9.0% on the Common Voice dataset [26] and 10.1% on the FLEURS dataset [27]. Transcripts of fairy-tale narration were further analyzed using the natural language processing tool stanza [28] to extract the lexical and syntactic information. Comprehensive details on speech transcription and annotation, including testing of algorithms’ accuracy, have been published previously [22].

### Language analysis

**Fig. 1:**
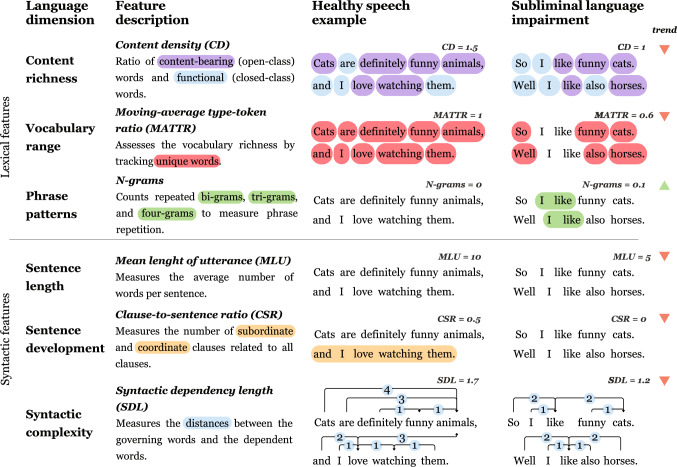
Description of language features used in discourse analysis

The criteria for language feature selection were as follows: (i) demonstrated sensitivity to linguistic abnormalities in PD, (ii) the capacity to cover complex language profile, each requiring distinct computational principles, and (iii) the feasibility of achieving fully automated analysis through advanced natural language processing techniques. Following our criteria, we selected 3 lexical and 3 syntactic representative features (Fig. 1). Including only a limited number of language features also decreased the probability of Type I error.

Lexical features included (i) content richness, assessed using *content density,* which reflects a potential tendency to prioritize or neglect content-bearing words over functional words that serve grammatical or syntactic roles, thereby altering the distribution of meaningful content and linguistic function. It is computed as the ratio of open-class words to closed-class words [22]; (ii) vocabulary range, evaluated with a *moving-average type-token ratio (MATTR)* [29], quantifying lexical diversity. MATTR is computed by applying function F, which iterates through the text using a fixed window size and a step size of 1. It calculates the ratio of unique words within the current window to the total number of words in that window. The final score is derived by averaging the resulting values. The window size was set to 84 words following our available dataset and prior suggestions for assessing the subject’s vocabulary; (iii) phrase patterns, evaluated using *n-grams* [22], which count the repetition of two-, three- and four-grams, i.e., following the participant’s usage of the exact phrases versus thinking of new ones, or the necessity to repeat the exact phrase multiple times before continuing in the narration.

Syntactic features included (i) sentence length, assessed by mean length of utterance [30], which counts the mean number of words in the sentence; (ii) sentence development, assessed using clause-to-sentence ratio [31], defined as the number of subordinate and coordinate clauses divided by the total number of sentences; (iii) syntactic complexity, assessed using syntactic dependency length, calculated as a sum of the number of words that intervene between the governing element (e.g., a verb) and the dependent element (e.g., an object or subject) calculated for each word in a sentence. The score in each sentence is normalized by the number of words in the sentence. The final score is the average.

### Statistical analysis

According to the normality of data after the Kolmogorov–Smirnov test, we used a one-way analysis of variance followed by Fisher’s least significant difference post hoc test or the Kruskal–Wallis test followed by the Mann–Whitney *U* post hoc test to analyze group differences at baseline. We employed paired t-test or Wilcoxon signed-rank test for each group, treating language features or clinical scales as the dependent variable, to evaluate changes between baseline and follow-up assessments. Pearson’s or Spearman’s correlation was used to analyze the relationship between changes in language features and clinical scales. The changes in parameters were computed as the difference between the score at the follow-up and the score at the baseline assessment. We addressed multiple comparisons via the Bonferroni adjustment for primary speech outcomes according to the three lexical/syntactic features and determined an estimate of *p* < 0.0167 (0.05/3).

## Results

### Participants

A total of 109 de-novo PD patients and 68 healthy controls were recruited. All subjects completed the 12-month follow-up; 92 PD patients were on stable dopaminergic medication (PD-treated), while 17 PD patients remained without dopaminergic medication (PD-untreated) (Table 1). PD-untreated patients were not initiated on dopaminergic treatment due to low and subjectively inobtrusive degree of symptom severity in line with their low MDS-UPDRS III motor score and, therefore, served as a control PD group representing natural disease progression. According to the MDS-UPDRS III speech item at the baseline, 46 PD patients (42%) demonstrated no speech impairment (score of 0), 62 PD patients (57%) mildly affected speech (score of 1), and 1 PD patient (1%) moderately affected speech (score of 2). After 12-month follow-up, PD-treated patients improved on MDS-UPDRS III, including all subscales, and STAI-X1. In contrast, the PD-untreated group further worsened on MDS-UPDRS III, including bradykinesia and rigidity subscores, and STAI-X1. No other changes between PD groups were detected. The control group improved performance on the MoCA score. The levodopa equivalent of the PD-treated group at the 12-month follow-up was 423 (SD 219) mg/day.

**Table 1:** Demographic and clinical data

	Healthy controls (*n* = 68)		PD-untreated (*n* = 17)		PD-treated (*n* = 92)		*p*-value
	Baseline	12-month follow-up	Baseline	12-month follow-up	Baseline	12-month follow-up	
General							
Age (years)	60.0 (9.2)		65.8 (15.4)		60.5 (12.1)		0.31
Gender (male)	44 (65%)		14 (82%)		57 (62%)		0.27
Education (years)	16.0 (3.5)		15.6 (3.1)		15.0 (3.1)		0.16
Disease duration^$^ (years)	-		3.0 (2.8)		2.0 (1.9)		0.18
Motor symptoms							
MDS-UPDRS III score	3.7 (3.1)	4.0 (3.2)	17.7 (6.9)^***^	25.2 (8.5)^***^	30.2 (11.6)^***^	26.6 (10.0)^***^	< 0.001^a,b,c^
Bradykinesia score	2.1 (2.0)	2.2 (2.1)	9.1 (4.4)^*^	12.8 (5.9)^*^	16.2 (7.2)^**^	14.4 (6.0)^**^	< 0.001^a,b,c^
Rigidity score	0.1 (0.5)	0.1 (0.4)	1.5 (1.5)^*^	2.8 (2.1)^*^	3.9 (2.7)^***^	3.1 (2.9)^***^	< 0.001^a,b,c^
Tremor score	0. (1.3)	1.0 (1.6)	4.0 (2.5)	5.5 (4.0)	6.1 (3.9)^*^	5.4 (4.0)^*^	< 0.001^a,b^
PIGD score	0.2 (0.4)	0.2 (0.4)	1.2 (1.3)	1.8 (1.6)	1.8 (1.7)^*^	1.6 (1.8)^*^	< 0.001^a,b^
UPDRS-III speech item	0.07 (0.26)*	0.18 (0.38)*	0.41 (0.51)	0.71 (0.59)	0.62 (0.51)	0.65 (0.56)	< 0.001^a,b^
Cognitive symptoms							
MCI presence	0 (0%)	0 (0%)	2 (12%)	2 (12%)	14 (15%)	16 (17%)	0.004^a^
MoCA total	26.4 (2.0)^**^	27.5 (1.9)^**^	25.5 (2.9)	25.6 (4.4)	25.3 (3.1)	25.1 (3.0)	0.18
MoCA language	2.8 (0.5)^*^	3.0 (0.2)^*^	2.4 (1.1)	2.5 (1.3)	2.6 (1.0)	2.6 (1.1)	0.051
Verbal fluency (words/minute)	17.6 (4.5)	18.5 (5.0)	17.1 (6.4)	18.1 (3.8)	17.2 (6.0)	16.9 (5.2)	0.89
Neuropsychiatric symptoms							
BDI-II	3.6 (4.0)	3.0 (3.2)	5.2 (6.7)	7.9 (6.8)	8.7 (5.3)	8.3 (5.4)	< 0.001^b,c^
STAI-X1	29.1 (6.8)	28.3 (6.8)	31.5 (5.8)^*^	37.6 (8.3)^*^	40.2 (9.8)^**^	36.8 (8.5)^**^	< 0.001^b,c^
STAI-X2	31.8 (7.4)	29.9 (8.2)	33.7 (6.7)	38.1 (7.1)	40.0 (9.5)	39.0 (8.6)	< 0.001^b,c^

The data are presented as mean (standard deviation) with p-values analyzed using analysis of variance/Kruskal–Wallis test, or as number (%) with p-values analyzed using the Chi-square test. Differences between baseline and the 12-month follow-up, analyzed using the paired t-test or Wilcoxon signed-rank test, are indicated as ^*^p < 0.05, ^**^p < 0.01, and ^***^p < 0.001

^a^ Significant difference between healthy controls and PD-untreated

^b^ Significant differences between healthy controls and PD-treated

^c^ Significant differences between PD-treated and PD-untreated

^$^ Disease duration was estimated from the self-reported occurrence of the first motor symptoms

*PD* Parkinson’s disease, *MDS-UPDRS* movement Disorders Society-Unified Parkinson’s Disease Rating Scale, *PIGD* postural instability/gait disorders, *MCI* mild cognitive impairment, *MoCA* Montreal Cognitive Assessment, *BDI* beck depression inventory, *STAI* State-Trait Anxiety Inventory

Out of 92 PD-treated patients, 69 who received monotherapy were included in the secondary analysis. Among them, 37 were treated with dopamine agonist ropinirole (PD-treated DA), while 32 received levodopa-carbidopa (PD-treated L-dopa). The remaining 23 PD-treated patients were on combination therapy, with the following treatment distributions: 8 received ropinirole combined with levodopa-carbidopa, 7 ropinirole combined with selegiline, 2 levodopa–carbidopa combined with amantadine, 2 selegiline alone, 1 pramipexole, 1 rotigotine, 1 levodopa-carbidopa combined with rotigotine, and 1 levodopa-carbidopa combined with amantadine and ropinirole. The main clinical differences at baseline included older age, lower MoCA score and higher MDS-UPDRS III postural instability and gait disorder, STAI-X2, and BDI-II scores in the PD-treated L-dopa group compared to the PD-treated DA group (Table 2).

**Table 2:** Demographic and clinical data for Parkinson disease group treated with L-dopa and with dopamine agonist

	PD-treated DA (*n* = 37)		PD-treated L-dopa (*n* = 32)		*p*-value
	Baseline	12-month follow-up	Baseline	12-month follow-up	
General					
Age (years)	53.3 (15.4)		69.7 (12.1)		< 0.001
Gender (male)	27 (73%)		17 (53%)		0.14
Education (years)	15.7 (3.1)		14.3 (3.1)		0.081
Disease duration^$^ (years)	1.7 (1.3)		2.1 (2.6)		0.84
Motor symptoms					
MDS-UPDRS III score	29.5 (10.3)^*^	27.6 (9.5)^*^	33.0 (12.0)^**^	26.4 (10.1)^**^	0.25
Bradykinesia score	16.1 (6.5)	15.5 (5.5)	17.4 (7.4)^**^	14.0 (5.9)^**^	0.59
Rigidity score	4.5 (3.0)	4.1 (3.6)	3.6 (2.5)^**^	2.3 (2.0)^**^	0.24
Tremor score	5.2 (3.4)	4.5 (3.7)	7.1 (4.2)	5.8 (4.3)	0.065
PIGD score	1.5 (1.3)	1.2 (0.9)	2.7 (2.1)	2.4 (2.5)	0.008
UPDRS-III speech item	0.57 (0.50)	0.62 (0.55)	0.72 (0.52)	0.69 (0.59)	0.25
Cognitive symptoms					
MCI presence	5 (14%)	4 (11%)	7 (22%)	12 (38%)	0.55
MoCA total	26.0 (2.8)	26.0 (2.5)	24.0 (3.1)	24.5 (3.4)	0.006
MoCA language	2.6 (0.5)	2.8 (0.4)	2.4 (1.0)	2.4 (0.9)	0.49
Verbal fluency (words/minute)	18.2 (5.9)	17.2 (3.8)	15.7 (6.1)	16.1 (5.2)	0.12
Neuropsychiatric symptoms					
BDI-II	6.8 (4.3)	7.1 (5.1)	10.4 (5.8)	9.3 (5.4)	0.005
STAI-X1	39.6 (7.9)^*^	35.7 (8.0)^*^	41.1 (10.8)	37.2 (7.6)	0.51
STAI-X2	38.0 (7.4)	37.0 (8.0)	42.4 (10.2)	42.3 (8.0)	0.044

The data are presented as mean (standard deviation) with *p*-values analyzed using independent *t*-test/Mann–Whitney *U* test, or as number (%) with *p*-values analyzed using the Chi-square test. Differences between baseline and the 12-month follow-up, analyzed using the paired *t*-test or Wilcoxon signed-rank test, are indicated as ^*^p < 0.05, ^**^p < 0.01, and ^***^p < 0.001

^$^ Disease duration was estimated from the self-reported occurrence of the first motor symptoms

*PD* Parkinson’s disease, *DA* dopamine agonists, *MDS-UPDRS* Movement Disorders Society-Unified Parkinson’s Disease Rating Scale, *PIGD* postural instability/gait disorders, *MCI* mild cognitive impairment, *MoCA* Montreal Cognitive Assessment, *BDI* Beck Depression Inventory, *STAI* State-Trait Anxiety Inventory

### Spoken language analysis

**Fig. 2:**
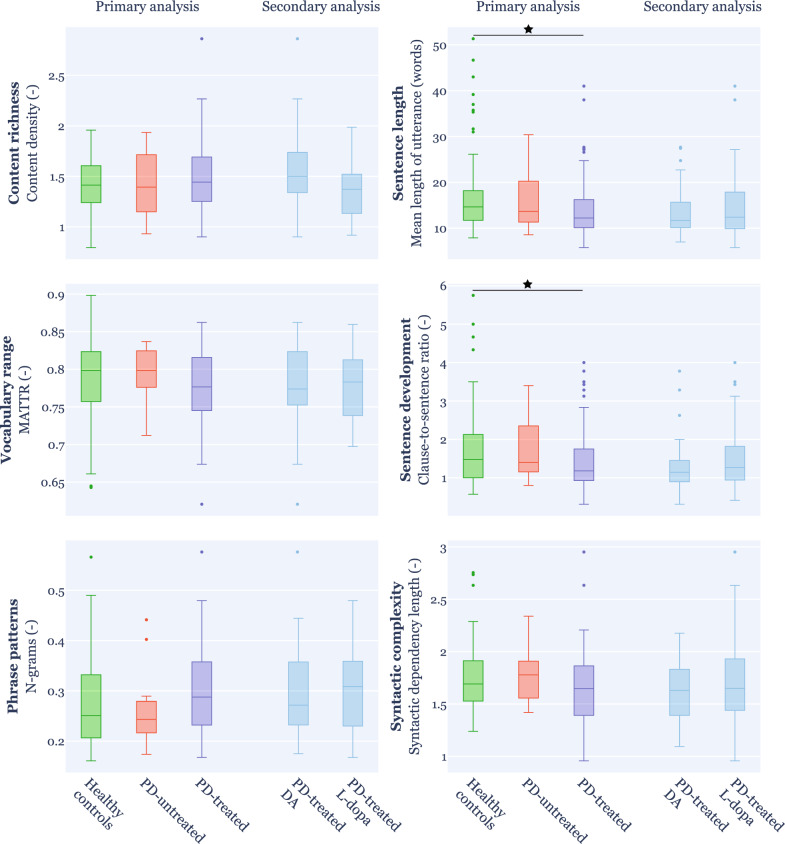
Group differences between individual language dimensions at baseline. The horizontal line represents the median, the box lower and upper quartiles, the bars minimum and maximum values that are not outliers, and the circles are outliers. Primary analysis was conducted on healthy controls, PD-untreated, and PD-treated groups. Secondary analysis was conducted on healthy controls, PD-untreated, PD-treated DA, and PD-treated L-dopa groups. Statistically significant differences between groups after Bonferroni’s adjustment are shown with *p < 0.05. *PD* Parkinson’s disease, *DA* dopamine agonists, *MATTR* moving-average type-token ratio

At the baseline, the primary analysis among the healthy control, PD-treated, and PD-untreated groups showed worse performance in PD-treated compared to healthy controls in sentence length (*p* = 0.018) and sentence development (*p* = 0.042) (Fig. 2). No feature was able to significantly discriminate between PD-treated and PD-untreated and between PD-untreated and healthy controls. The secondary analysis among the healthy control, PD-untreated, PD-treated DA, and PD-treated L-dopa groups revealed no differences between groups.

**Fig. 3:**
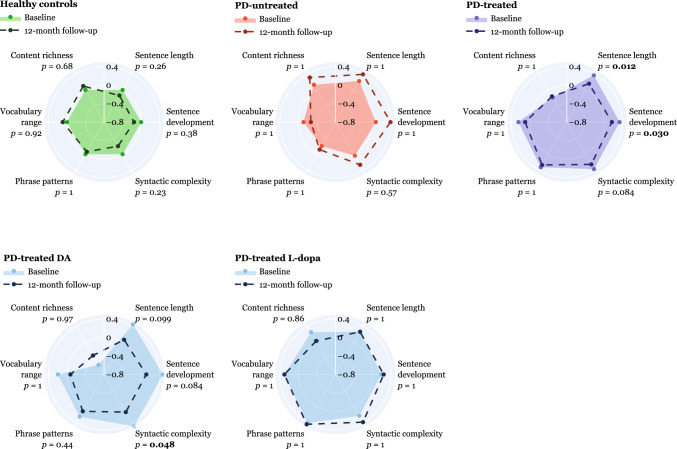
Longitudinal changes in individual language dimensions after 12-month follow-up. Dots with filled areas across spider plots demonstrate the *z*-scored values of individual dimensions at baseline; dots with dash lines represent the *z*-scored values at 12-month follow-up. Higher values indicate greater severity. *p*-values after paired *t*-test/Wilcoxon signed-rank test are shown after Bonferroni’s adjustment. *PD* Parkinson’s disease, *DA* dopamine agonists, *MATTR* moving-average type-token ratio

At the 12-month follow-up, the PD-treated group showed significant improvements in syntactic features, particularly in sentence length (*p* = 0.012) and sentence development (p = 0.030) (Fig. 3). No changes were registered in the lexical features for the PD-treated group. No change in language features for the PD-untreated and healthy control group was seen. In the secondary analysis, the PD-treated DA group manifested significant improvement in the syntactic feature of sentence length (*p* = 0.048), while no improvement was detected in the PD-treated L-dopa group.

### Relation between change in language and clinical scales over time

In the PD-treated group, significant correlations were observed between changes in verbal fluency and phrase patterns (*r* = 0.29, *p* = 0.042). No correlations were found between changes in language parameters and levodopa equivalent intake, MoCA total score, the MDS-UPDRS III scale/subscales, including the MDS-UPDRS III speech item, BDI-II, STAI-X1, and STAI-X2. In the PD-treated DA and PD-treated L-dopa groups considered individually, no relationship was observed between changes in language parameters, age, and changes in clinical scales.

## Discussion

Based on the language analysis during spontaneous fairy-tale narration in a large sample of de-novo PD patients, this observational, naturalistic study reports for the first time the positive effect of long-term levodopa therapy on spoken language. This language improvement cannot be interpreted as a simple effect of intraspeaker variability during the time because speech performance in the PD untreated control group did not change or even tended to deteriorate over 12 months due to natural disease progression. Although previous evidence about discourse-level impairments in PD is limited, our finding is in principal accordance with the pilot studies reporting the short-term ameliorating effect of levodopa on language function assessed in particular via verbal fluency [9–11]. Previous research also showed that long-term L-dopa medication in untreated PD improved frontal-subcortical and posterior cortical cognitive functioning, including improvement in confrontation naming and verbal fluency [12]. In addition, one study found no significant narrative discourse deficits in 18 PD-treated individuals with preserved cognitive function [32], indirectly supporting our findings that dopamine replacement improves language function.

Among language production deficits in PD, word-finding difficulties are commonly reported [33], along with challenges in naming objects, generating words fluently, constructing syntactically complex sentences, and maintaining coherence and informativeness in speech [34, 35]. The presence of language deficits is more frequent among PD patients with MCI [36]. However, only 15% of our cohort of de-novo PD patients clinically manifested MCI. This likely contributed to the fact that we found deficits only in the syntax domain while the lexical domain remained mostly unaltered. These findings align with a previous study, which reported no significant difference in the lexical domain in early-stage PD patients without MCI compared to healthy controls [22]. Accordingly, after a 12-month follow-up, the PD-treated group showed improvements specifically in syntactic dimensions, including sentence length and sentence development. This reflects an enhanced ability to produce longer, more complex sentences with coordinate and subordinate clauses.

Interestingly, the beneficial effects of dopaminergic therapy on syntactic aspects of language were more pronounced in PD patients receiving dopamine agonist monotherapy compared to those treated with levodopa. One explanation may be the selective capacity of dopamine agonists to enhance executive functions, motivation, and reduce depressive symptoms and apathy. Dopamine agonists directly stimulate dopamine receptor subtypes (D2, D3) in frontal and limbic regions linked to these cognitive and motivational processes, potentially supporting language functions more effectively than levodopa. Dopamine agonists have also been shown to improve working memory performance in newly diagnosed PD patients [37], suggesting targeted effects on dorsolateral frontostriatal circuits. While previous research indicated both levodopa and dopamine agonists could transiently enhance cognitive performance [38], dopamine agonists may more effectively address neuropsychiatric symptoms such as depression, anxiety, and apathy [39]. Although the dopamine agonist group in our study was significantly younger than the levodopa group, we found no direct relationship between age or neuropsychiatric changes and language improvements. Thus, further research is necessary to clarify the potential specific benefits of dopamine agonists on language function.

The impact of dopamine-replacement therapy on high-level language functions remains unclear since it may affect different cognitive processes in various ways [40]. Cognitive functions such as working memory support higher-level, real-time organizational aspects of discourse. Improvement in working memory function observed on dopaminergic medications [41] may thus underlie the normalization of language function observed in our current study. Yet, we did not observe any changes in the total MoCA score or its language subscore following dopamine replacement, implying that linguistic discourse analysis may be a more sensitive measure of the cognitive effects of dopamine therapy in PD than the MoCA test. Syntactic language abilities mainly rely on the proper functioning of Broca’s area [42] as well as on the integrity of the tract connecting this region to the striatum [43, 44]. A previous study found that compared to PD patients on levodopa treatment, PD patients without levodopa exhibited increased cerebral activation in the left hemisphere, mainly in the frontal and parieto-occipital areas, suggesting they had to rely on neighboring brain areas to compensate for impaired regions involved in language processing [45]. Conversely, the normalization of striatal activation was observed in PD patients after levodopa treatment, implying that levodopa may reduce the need for compensatory activation in the parieto-occipital areas. Furthermore, the left caudate nucleus, a part of the striatum, has been shown to be activated only during syntactic processing [46], and the integrity of the tract connecting Broca’s area to the left caudate head is crucial to perform syntactic tasks accurately [47]. No such anatomic-clinical association was observed in speech tests involving morphological or phonological tasks. Also, the left caudate nucleus activation in fMRI was observed in PD patients on levodopa treatment compared to PD patients without levodopa [45].

There has been an ongoing debate about whether and to what extent changes in discourse observed in PD are due to non-cognitive factors, particularly dysarthria. The improvement in language cannot be solely attributed to changes in motor speech function, as we found no significant changes in the MDS-UPDRS III speech item following dopamine therapy, nor any correlation between changes in language variables and the MDS-UPDRS III speech item as well as the total score. Indeed, a previous study showed that motor speech improvement after long-term levodopa treatment is phenotypically specific to PD patients manifesting with predominant dysphonia [48]. The majority of our PD sample has only mildly affected speech or demonstrated no speech impairment. More severe motor speech impairments could have a greater effect on discourse measures [49]. In addition, we did not detect a link between the improvement of language and the extent of levodopa equivalent. We were also not able to verify the hypothesis of common pathophysiology between gait and language dysfunction linked to dopamine deficits [13], as all MDS-UPDRS III subscales showed a similar extent of improvement after one year of levodopa initiation and no correlation between change in language features and postural instability/gait disorders subscore was seen.

Regarding cognitive clinical scales, we observed a correlation between verbal fluency and phrase patterns, indicating that verbal fluency reflects particularly the lexical domain of language skills. Indeed, a previous study reported that verbal fluency heavily relies on the vocabulary knowledge of patients [51]. Another study showed that PD patients off medication used more high-frequency verbs than controls, indicating that dopamine plays a role in the normal functioning of the lexico-semantic network for verbs [10]. However, our findings on relationships between changes in verbal fluency and lexical language parameters should be interpreted cautiously, as lexical deficits in our PD cohort did not significantly differ from controls.

Automated language analysis holds strong potential for clinical implementation. Our fully automated pipeline eliminates subjective rater bias and ensures objective, quantitative metrics. Although severe dysarthria in movement disorders can complicate speech analysis and transcription, linguistic features in the present study remain highly robust to transcription errors. The misrecognized words often retain their linguistic properties—for example, if *cat* is transcribed as *bat*, the word type remains, and the word may still contribute to lexical diversity. This robustness has already been demonstrated in PD [22] and even in multiple system atrophy [49], where dysarthria is typically more severe. Our approach requires only 90-s narrative recordings and leverages freely available natural language processing tools, minimizing patient burden and assessment costs. Therefore, language assessment has a potential to be considered a practical biomarker for disease monitoring and treatment response in routine care, enabling timely intervention. A key future advantage of analyzing language via spontaneous speech is its potential for robust continuous assessment via smartphones in real-world settings [52]. This approach could offer greater sensitivity to therapeutic interventions by leveraging extensive longitudinal data collected in a natural environment.

This study has potential limitations. We detected improvement in cognitive performance via MoCA over time only in the healthy control group. This observation could be explained by the practice effect, which has been reported among healthy older adults, where improvements in repeated MoCA tests were particularly observed between the first and second administration [53]. We observed only a tendency towards worsening in linguistic features in the PD-untreated group. These findings might be attributed to the smaller sample size of the PD-untreated group due to the small number of patients who did not require dopamine medication in the first year of our longitudinal study. Future research should also perform more rigorous neuropsychological testing together with language assessment.

In conclusion, our findings provide evidence that long-term dopaminergic treatment has a positive impact on high-level language abilities in de-novo PD patients, specifically enhancing syntactic processing. Notably, these improvements are not driven by motor speech enhancement, underscoring the role of dopamine replacement in cognitive-linguistic processing. As language deficits can profoundly impact daily life and social interactions, automated analysis of spontaneous speech using natural language processing may offer an effective method for predicting and monitoring linguistic abnormalities in PD and support more targeted therapeutic approaches. Future imaging studies using multi-parametric brain magnetic resonance imaging could provide additional insights into the neurobiology of language abnormalities in PD.

## Data Availability

Individual participant data that underlie the findings of this study are available upon reasonable request from the corresponding author. The data are not publicly available due to their containing of information that could compromise the privacy of study participants.
